# Prioritizing quality of geriatric rehabilitation from the older adults’ perspective: A nominal group technique study

**DOI:** 10.1177/02692155251404003

**Published:** 2025-12-04

**Authors:** Anne L Lubbe, Wim G Groen, Marjon van Rijn, Deborah C Mittelmeijer, Cees MPM Hertogh, Bianca M Buurman, Margriet C Pol

**Affiliations:** 1 Departement of Medicine for Older People, 1209 Amsterdam UMC, Vrije Universiteit Amsterdam, Amsterdam, The Netherlands; 2 Aging & Later Life, Amsterdam Public Health, Amsterdam, The Netherlands; 3 Vivium Zorggroep, Naarden, The Netherlands; 4 Ageing & Vitality, Rehabilitation & Development, Amsterdam Movement Sciences, Amsterdam, The Netherlands; 5 Center of Expertise Urban Vitality, Faculty of Health, Amsterdam University of Applied Sciences, Amsterdam, The Netherlands; 6 Department of Physiotherapy, Faculty of Health, 98812 Leiden University of Applied Sciences, Leiden, The Netherlands; 7 Research group occupational therapy: Participation and Environment, Faculty of Health, Center of Expertise Urban Vitality, Amsterdam University of Applied Sciences, Amsterdam, The Netherlands

**Keywords:** Geriatric rehabilitation, older adult prioritization, quality, nominal group technique

## Abstract

**Objective:**

To identify and prioritize aspects of quality in geriatric rehabilitation from the perspective of older adults.

**Design:**

Qualitative study using a structured Nominal Group Technique.

**Setting:**

Three nominal group technique sessions were conducted in geriatric rehabilitation settings in the Netherlands.

**Participants:**

We included older adults admitted to geriatric rehabilitation, Dutch proficiency, the ability to communicate and engage in group discussions, and an intention to return to independent living.

**Intervention:**

Participants engaged in structured nominal group technique sessions. Each session included five steps: introduction, idea generation, exchange of ideas, discussion, and ordering and ranking

**Main measures:**

Audio-recorded sessions were transcribed verbatim and analyzed using a combined inductive and deductive coding. Quality aspects were prioritized based on their summed ranking scores, relative importance, and selection frequency.

**Results:**

Eighteen older adults were included. Participants identified five key priorities for quality in geriatric rehabilitation: (1) good preparation with clear expectations, (2) well-organized therapy and care, (3) relationships with healthcare professionals that acknowledge individual needs, (4) clear, respectful communication, and (5) autonomy in treatment decision-making. The qualitative analysis supported these priorities with three overarching themes: the value of a supportive environment, feeling heard and understood, and the need for guidance and involvement.

**Conclusion:**

This study provides priorities for improving quality in geriatric rehabilitation, according to the experiences of older adults and offers direction for implementation in clinical practice.

## Introduction

Geriatric rehabilitation faces increasing pressure due to staff shortages, limited bed availability, and an ageing population.^1^ Shorter lengths of stay further limit the time for effective rehabilitation,^2,3^ potentially compromising care quality for older adults.^4^ In response, maintaining high-quality care is more critical than ever. Geriatric Rehabilitation is defined as “a multidimensional approach of diagnostic and therapeutic interventions, the purpose of which is to optimize functional capacity, promote activity and preserve functional reserve and social participation in older people with disabling impairments.”^5^ There are various definitions of older adults. In this study, we considered older adults as all individuals eligible for geriatric rehabilitation. Geriatric rehabilitation is not solely determined by chronological age but rather by diagnosis, with the primary goal of enhancing independent functioning and facilitating discharge to home.^6–8^

International frameworks support quality measurement in geriatric rehabilitation,^5^ such as Verenso's 7-point performance indicator scale, which assesses domains including effectiveness, patient experience, professional expertise, and integrated care.^9^ However, these tools often rely on predefined indicators that may overlook the perspectives of older adults. This raises concerns about whether current metrics truly capture what matters most to patients, especially when clinical outcomes are prioritized over personal experiences.

Older adults value clear information, autonomy, involvement in decision-making, structured care, and social engagement during rehabilitation.^10,11^ These factors influence both satisfaction and outcomes, such as length of stay and discharge timing.^2,12^ Yet, patient perspectives remain underrepresented in quality assessments, despite their ethical and practical relevance. Sole reliance on clinical indicators provides an incomplete view of care quality.^5,9^

Integrating the views of older adults into quality measurement is essential not only for person-centered care but also for supporting effective implementation of improvements. However, it remains unclear which aspects of care older adults prioritize most, and whether current measurement frameworks accurately capture these priorities.

Setting the right priorities is essential for successful implementation in a practice currently under high demand. Despite being evidence-based, many implementation efforts fail because real-world practice is influenced by a range of complex factors, such as unrealistic expectations and behavioral and organizational change.^13,14^ Staying close to the experiences of older adults and allowing those receiving care to identify what matters most helps ensure that implementation efforts are grounded in actual needs.

The aim of this study is to identify and prioritize quality-related topics of geriatric rehabilitation from the perspective of older adults, using a Nominal Group Technique. Prioritization is needed to facilitate the practical implementation of improvements, recognizing that addressing all topics at once is neither feasible nor effective. Involving older adults in this process ensures that implementation efforts focus on what matters most to them.

## Methods

The Medical Ethics Committee of The Amsterdam UMC has confirmed that the regulations under the Medical Research Involving Human Subjects Act do not apply to this study (2023.0284). Written informed consent for participation was obtained from participants before the start of the group interview.

We used the Nominal Group Technique. This is a mixed methods approach based on structured group discussion, often used in situations that require complex problem-solving, decision-making, priority setting, and consensus reaching.^15,16^ The nominal group technique was developed by Delbecq and Van de Ven as a process for “*identifying strategic problems and developing appropriate and innovative programs to solve them*.”^16^ The nominal group technique facilitates the generation of ideas about problems, solutions, or both, which are then discussed and ranked in order of priority by individual participants.^17,18^

We used the nominal group technique method for its ability to promote and facilitate discussion and voting around certain complex problems. The nominal group technique method was particularly suitable to prioritize the quality of geriatric rehabilitation from the older adult's perspective,^19^ as it enabled the direct involvement of older adults themselves, whose experiences and views are essential in shaping care that meets their needs.

The nominal group technique method encompasses five steps: 1: introduction, 2: idea generation, 3: exchange of ideas, 4: discussion, and 5: ordering and ranking (see Table 1).^18^ The following figure provides a detailed description of each step, including the instructions given to the participants.

**Table 1.** Guide nominal group technique.

Nominal group technique method	
Step 1 Introduction	The introductory step briefly outlines the meeting and establishes agreement on the process. *Instruction*: “We will spend approximately two hours together, working through five phases. The key question we aim to address today is: What are the essential topics of high-quality rehabilitation?”
Step 2 Idea generation	During this step, each participant is provided with a paper to document their ideas on the quality of geriatric rehabilitation from their perspective. *Instruction*: “You will work individually. Each of you will receive a paper on which you can reflect and write down what high-quality rehabilitation means to you.”
Step 3 Exchange of ideas	Participants share their ideas verbally, with an observer documenting each suggestion. After all participants have shared their ideas, we present the themes from previous research ^10,11^. *Instruction*: ‘’We will then record everything you have written on a large board. Each person will take turns sharing one of their ideas until no new contributions are made.’’
Step 4 Discussion	Participants engage in discussions to explore emerging ideas. *Instruction:* ‘’Next, we will discuss the ideas that have been shared. You can ask each other questions, introduce new ideas, combine, or group existing ones. This will be an open conversation, and we will allow everyone to speak. So everyone could have their own priority on the ideas for the next step. After this discussion, we will take a short break where you can have something to drink. While you take a break, we will document the ideas as thoroughly as possible.’’
Step 5 Ordering and ranking	The ideas are organized and ranked on paper, and ranked for importance from most to least significant. *Instruction*: ‘’After the break, you will receive the identified ideas on paper. You can arrange them in order of importance, starting with the idea that is most important to you and continuing downward to the least important idea.’’

The study population consisted of participants prospectively recruited from four geriatric rehabilitation centers in the northwest of The Netherlands. These centers are affiliated with the University Network of Care Organisations for older adults (UNO) Amsterdam. We applied the following inclusion criteria: older adults in geriatric rehabilitation, admitted with an indication for geriatric rehabilitation, proficient in Dutch, able to communicate and consider themselves capable of participating in a group conversation, and intending to return to an independent living situation after admission to geriatric rehabilitation. The treating physician determined the eligibility of participants for inclusion based on the inclusion criteria.

The nominal group technique protocol for this study was pilot-tested with a group of four older adults to test its suitability and feasibility for this population.^20^ The pilot testing ensured the protocol was realistic and that older adults could easily understand the questions. While most steps in this pilot phase worked well, the ranking process, where participants assigned scores to prioritize ideas, proved challenging, as they rated everything as equally important. To address this, we adapted the ranking procedure by introducing a more visual method, allowing participants to physically move and rank topics to force the creation of a list of priorities.

We used a purposive sampling approach to ensure the inclusion of a diverse group of participants varying in sex (male/female), age (years), and diagnosis (stroke, elective surgery, trauma, amputation or other^21^).^22^ The healthcare professionals responsible for participant recruitment were elderly care physicians and (physio)therapists affiliated with the participating geriatric rehabilitation centers. They provided eligible participants with information about the study, including its objectives and procedures. If an older adult agreed to participate and provided informed consent, the researcher contacted the participant to offer further information and address any questions. The attending physician determined eligibility. Subsequently, an appointment was made for the group interview. Additionally, as an alternative recruitment route, posters were placed in the rehabilitation centers. Older adults could then contact the researcher directly, after which their treating physician evaluated their eligibility. All participants provided both written and verbal informed consent prior to participation.

### Data collection

The nominal group technique meetings were chaired by the first author (AL) and supported by DM (observer/research assistant). Data were collected from October 2023 to January 2024, by AL and DM. The meetings followed a structured format comprising five steps (see Table 1). All sessions were audio-recorded and transcribed verbatim. Data on participants’ sex, age, and admission diagnosis were also collected.

After piloting, we conducted three nominal group technique interviews at three geriatric rehabilitation centers in the Netherlands. The quality themes proposed at the end of Step 3 were derived from a comprehensive scoping review^10^ and interview study.^11^

During the nominal group technique interviews, the moderator (AL), who had no treatment relationship with the participants, facilitated the discussions. An observer (DM) was also present.

### Data analysis

We used two approaches to ensure the content and face validity of the emerging ideas: qualitative content analysis of discussion transcripts, and collation and scoring of the individually ranked items.^23^

Group discussion transcripts (obtained in Step 4) were analyzed to provide contextual depth, capturing the rationale for individual ideas and the related discussions that occurred during the clarification step.^24^ Thematic analysis was used to systematically identify, and interpret patterns within the data.^25^ The analysis followed the six phases as described by Braun and Clarke.^25^ The nominal group technique sessions were transcribed verbatim and read by the first author and DM (phase 1: familiarization with the data). Inductive open coding was applied to code the transcripts (phase 2: generating initial codes). Codes were systematically clustered into potential themes (phase 3: searching for themes), which were then reviewed (phase 4: reviewing themes), redefined together with the research team (phase 5: defining and naming themes), and finally written up (phase 6: producing the report).

We used three nominal group technique sessions to prioritize the quantitative part (Step 5). During the group discussion, participants were able to refine individual ideas and assign their own priorities to these ideas. The ideas from each nominal group technique session were taken to the following group discussion and were ranked there in order of perceived importance. The scores from the three sessions were summed. The item with the highest priority received a score of 15, and the item with the lowest priority a score of 1. The three group discussions created a top five of the most essential ideas based on their relative importance (sum scores) and the frequency of each item scoring.^18^ Subsequently, the voting frequency for an item being most important was determined to assess how often a particular theme was voted for, indicating its popularity among participants.^18^ The final conclusion was drawn based on relative importance (the proportion (%) of all scores in the top five, which was calculated using the following equation: (score achieved for the item)/(maximum possible score) × 100), as this measure accounts for variations in group size.^18^

## Results

A total of 18 participants from three organizations participated in the three group discussions. The participants’ ages ranged from 63 to 91 years, with a mean age of 76 years. There were 11 female and 7 male participants. Participants had various diagnoses, including orthopedic (n = 9), internal (n = 3), neurological (n = 3), vascular (n = 1), and amputation (n = 2). The length of stay varied, as participants were still undergoing rehabilitation at the time of data collection. Table 2 provides an overview of participant demographics and group composition.

**Table 2.** Participants in the nominal group technique.

Code	Sex	Age (years)	Living situation	Diagnosis	Rehabilitation center	Days in rehabilitation
P1	M	83	Alone	Cardiac bypass	A	37
P2	F	68	Married	Guillain-Barré	A	23
P3	F	80	Alone	Femoral fracture	A	16
P4	F	83	Alone	Femoral fracture	A	5
P5	F	65	Alone	Tibia fracture	A	29
P6	F	78	Alone	Colostomy	A	35
P7	F	69	Alone	Sepsis	A	39
P8	M	86	Married	Deep venal thrombosis	B	38
P9	M	76	Alone	Costal fracture	B	16
P10	F	91	Alone	Total hip prosthesis	B	14
P11	F	64	With daughter	Total hip prosthesis	B	25
P12	M	70	Married	Amputation leg	B	21
P13	F	77	With son	Multiple Sclerosis	C	20
P14	M	63	Alone	Humerus fracture	C	43
P15	F	75	With son	Amputation leg	C	44
P16	F	77	Married	Shoulder prosthesis	C	41
P17	M	86	Married	Total hip prosthesis	C	77
P18	M	82	Married	Stroke	C	12

In step 1–3, participants generated and shared a broad range of ideas in response to the central question on what defines high-quality rehabilitation. After additional themes from prior research were introduced, 14 unique ideas reflecting diverse experiences emerged, which are listed in Table 2.

The group discussions from step 4 provided rich insights into older adults’ perspectives on high-quality rehabilitation. An analysis of their input revealed a layered structure of priorities. Initially, participants focused on more visible, day-to-day care. However, upon deeper examination of what truly matters, two additional underlying themes emerged. These findings were reinforced by the prioritization exercise (Step 5 of the nominal group technique). In total, three overarching themes were identified: (1) The importance of a comfortable and supportive environment in rehabilitation, (2) Feeling heard and understood: the role of trust and communication in rehabilitation, and (3) Finding my way: guidance, involvement, and support in rehabilitation. Each theme will be described and supported by quotes from participants.

### The importance of a comfortable and supportive environment in rehabilitation

Participants described how basic conditions, such as cleanliness, proper nutrition, appropriate room temperature, and furniture, strongly influenced their perception of care quality. They also highlighted the value of social interaction and structure in daily routines, which contributed to a sense of normalcy.

Hygiene emerged as a key issue, with varied experiences. Some felt empowered to request what they needed:

*‘You have to indicate when you want to take a shower; it's not automatically scheduled or anything. But well, I'm assertive enough to say that I want to shower.’ (P5). While others experienced limited opportunities to bath: ‘I think I've only showered once in the two weeks I've been here; the rest of the time, I used a washcloth.’ (P3)*.

Participants also spoke about the emotional comfort provided by a safe and familiar environment, shaped by both the presence of healthcare and the setting:

*When I came back the second time after being away. I went home for the first time this week for just two hours, and it felt really surreal after 12 weeks. But still, being back here felt like home because I feel safe here. (P6)*

### Feeling heard and understood: The role of trust and communication in rehabilitation

Communication with healthcare professionals was described as fundamental. Participants described communication at three levels: between the organization and the older adult, between the healthcare professional and the older adult, and among healthcare professionals themselves. They expressed concern about continuity and trust in the face of staff turnover and temporary personnel. As one participant stated:

*I find it very difficult because I see that personal approaches and communication breakdowns often occur due to staff turnover, part-time workers, or temporary staff. Or when it says ‘temporary staff’ on the board, they should still know what's happening with us in Ward 4. You start to wonder if the structure and continuity are maintained and if people know what's happening behind the door. (P11)*

Temporary staff and staff shortages were discussed in the various organizations. Despite these challenges, participants were understanding and respectful toward staff, recognizing their efforts under difficult circumstances. One participant said:

*‘I understand that the staff is busy […], but even so, you still receive only the minimum level of care.’ (P14). ‘You could say, “I don't care about that, I'm the patient, and I want this,” but if you look closely, you can see they're working themselves to the bone, but there is a lower limit.’ (P17) ‘No, you can't blame them; they're all doing their best.’ (P18)*

They appreciated moments of personal attention, noting that even a small gesture could have a significant impact as follows:

*Last night, she sat with me momentarily, which I appreciated. Then, you get feedback and talk to yourself because otherwise, it stays in your head. I really appreciate it when they sit on the bed with you, just giving attention, not standing, but sitting down for a moment. Then, you feel connected with the nursing staff. (P6)*

Participants highlighted personal connection as a key indicator of rehabilitation quality. Therefore, good communication is essential for the effective organization of rehabilitation, but also for the overall well-being of older adults.

### Finding my way: Guidance, involvement, and support in rehabilitation

For participants, the rehabilitation process begins with the provision of information in the acute phase before entering geriatric rehabilitation. While they were informed where they will undergo rehabilitation, they experienced little clarity about what rehabilitation actually entails. Conversations revealed that, especially in the first few days, uncertainty was experienced, with much of the necessary information having to be figured out independently. One of the participants expressed this challenge:

*It was mainly about the first few days here. I would have liked someone from the nursing staff to guide me through those difficult days. I really struggled in the beginning. (P11)*

This highlights the need for structured guidance at the start of rehabilitation, as participants experience a strong sense of dependency in this phase. As rehabilitation progresses, older adults wanted to be more involved in setting their rehabilitation goals and to gain insight into their recovery trajectory. Some participants emphasized the importance of this involvement:

*‘When you talk about high-quality rehabilitation, you expect that everything will be done to achieve that goal, and if that doesn't happen, of course, that's understandable.’ (P10) ‘As a patient, you really want to know where you're heading and what you will do about it. So, you can really gain insight yourself.’ (P11)*

In the rehabilitation process, older adults work towards their goals with support from healthcare professionals throughout the journey. Participants find help in areas where they cannot yet manage independently, but they also perceived assistance in the form of motivation and clarification.

*‘I also feel like I'm making progress, and they support me. She motivates me to keep going, to take that one small step forward. […] But we're focused on training, and she encourages me to do that. I want to address that point daily and experience it as very positive here. (P14)*

This quote underscores the essential role of the healthcare professional in the progress of the older adults and the support for both the physical and emotional aspects of rehabilitation.

Following the group discussions, participants were invited to prioritize the ideas in Step 5 of the nominal group technique. This step offered insight into which topics were considered most essential to high-quality rehabilitation from the perspective of older adults. While the group discussions underscored a broad and layered understanding of care experiences, the ranking exercise forced participants to make choices. The top-rated items correspond closely with the deeper themes described earlier, especially in the importance of communication, personal attention, and support in navigating the rehabilitation process. Table 3 presents the results of this prioritization.

**Table 3.** Nominal group technique ideas.

Ideas	Sum nominal group technique	Ranking (Sum nominal group technique)	Frequence voting	Ranking (Frequence voting)	Relative importance	Ranking (Relative importance)
Good preparation for rehabilitation, with clear expectations^a^^,^^b^^,^^c^	37	1	10	2	43.2	1
Well-organized therapy and care^a^	35	2	12	1	37.7	2
Forming relationships with healthcare staff with recognition of individual needs^a^^,^^c^	35	3	9	4	37.1	3
Clear, respectful, and effective interpersonal communication^a^^,^^b^	32	4	9	5	35.3	4
Autonomy in decision-making regarding involvement in treatment discussions^a^^,^^b^^,^^c^	28	5	10	3	32.3	5
Being able to tell the story^a^^,^^b^	16	6	5	7	17.6	6
I need information about the rehabilitation^a^^,^^b^^,^^c^	15	7	6	6	16.7	7
I receive help with what I cannot do yet and with learning to cope with it^a^^,^^b^	13	8	4	10	15.4	8
A comfortable environment that helps^a^^,^^b^	12	9	4	11	14.3	9
Hygiene is important^a^	11	10	5	8	11.8	10
Staff expertise is affected by their workload^a^	10	11	5	9	11.1	11
Daily activities, providing information about them, a clear schedule, and organization^a^	9	12	4	12	10.8	12
Good and sufficient nutrition (variety, quality)^a^	9	13	3	14	8.7	13
The opportunity to meet others and have visitors^a^^,^^c^	8	14	4	13	8.0	14

^a^Nominal group technique study, ^b^Scoping review,^10^ ^c^Interview study.^11^

Sum nominal group technique: the summing of votes.

Frequence voting: the frequency with which an idea appeared in the top five.

Relative importance: the proportion (%) of all scores in the top five, which was calculated using the following equation: (score achieved for the item)/(maximum possible score) × 100.

Table 3 shows that the top five ideas have significantly higher scores across all three analyses than the other nine ideas. The sum score and relative importance yield the same top five; however, the frequency with which an idea is ranked as a top priority alters the order within this top five. This additional value helps to determine whether a highly ranked idea is widely endorsed among participants. It is particularly useful when ideas have the same sum score.

The results show that the top five ideas stand out significantly across all three analytical methods (sum score, frequency voting and relative importance). While the differences within the top five are relatively small, there is a notable drop is scores between the fifth- and sixth-ranked ideas.

## Discussion

The aim of this study was to identify and prioritize quality-related topics in geriatric rehabilitation using a nominal group technique. Fourteen ideas were identified and prioritized, resulting in a five ideas being most prioritized from the perspective of older adults: (1) good preparation for rehabilitation, with clear expectations, (2) well-organized therapy and care, (3) forming relationships with healthcare staff with recognition of individual needs, (4) clear, respectful, and effective interpersonal communication and (5) autonomy in decision-making regarding involvement in treatment discussions.

Quality of care is often assessed from the perspective of healthcare professionals^26^ or through functional outcome measures,^5,9^ with limited attention given to the older adult's own perspective on what quality of care entails and what is most important.

This research method employed a structured approach to idea generation and prioritization, ensuring individual input and integrating existing research insights. By incorporating insights from previous research,^10,11^ a broad perspective was considered in the ranking process. However, insights from previous research were introduced only after participants had formulated their quality topics, ensuring they did not influence their personal idea generation. During the silent idea generation phase, themes from the two previous studies emerged, although in different words, with the same underlying meaning. This confirms that the group discussions reinforced the findings of these prior studies.^10,11^ This study adds the prioritization step.

The group discussions provided deeper insight into how participants perceived quality, revealing that the extent of the discussion did not always align with prioritization. During step 4, participants engaged in group discussions to share their perspectives on quality. Ideas were elaborated throughout these conversations, and individuals could prioritize the mentioned ideas in the final step (step 5). Notably, some topics were widely discussed in step 4 but did not receive high rankings. For instance, nutrition was frequently mentioned, yet it did not emerge as a top priority.

In contrast, workload presents a different perspective; participants extensively discussed the recognized workload and the limited time available to staff. However, this issue also did not rank highly, possibly because older adults observed that staff members are willing but lack sufficient time. While this does not necessarily affect the quality of care, it does impact the opportunity for personal interaction. This aspect is highly valued, as reflected in the top five priorities.

Building a strong therapeutic relationship is essential in rehabilitation, yet time constraints challenge healthcare professionals and older adults to achieve truly person-centered care. A stable team of healthcare professionals helps to build a relationship, but creating a therapeutic relationship takes time. In the interview study by Lubbe et al.,^11^ older adults observed a high workload, yet the importance of a human touch also emerged in the aforementioned results of the current study. Studies from the nurse perspective show, that there is a demonstrated desire for more person-centered care, with nursing staff also noticing a lack of time, as shown in our studies from the perspective of the older adults.^27^ Providing emotional support to older adults and families is seen as something important for nurses, but they often have only a limited opportunity.^27^ Additionally, giving comfort and having conversations with older adults were most frequently cited by nurses as missed care, followed by patient observation, education, and updating care plans.^28^

From the healthcare professional, we could see the lack of time for communication, but point 11 in the prioritization about the workload is not very high in the prioritization of older adults, while it's a large part of the group discussion during the nominal group technique interviews. Personal connection with the healthcare professional is the most important.

Another possible reason the participants did not prioritize this idea highly is the way it was formulated. Presented more as an observation with a negative connotation, it may have influenced participants’ interpretation differently.

Clear information and preparation are crucial in rehabilitation, as they shape expectations and empower older adults to set goals. The top five start with good preparation for rehabilitation and knowing what to expect. In home-based geriatric rehabilitation, people need education and information, especially in the early stages.^29^ Communication and the information provided contribute to older adults’ ability to set rehabilitation goals independently.^30^ Research shows that 15% of treatment outcomes are determined by expectations, making it understandable that older adults view clear expectations as essential for high-quality care.^31^ An important recommendation incorporating the older adult's perspective could be to show an informational video before rehabilitation.^32^

The individualized approach is reflected in the recently developed Generic Compass (Generiek Kompas) in the Netherlands,^33^ where the first foundational element is understanding the wishes and needs of the individual who requires care. This framework places the person at the center and extends beyond the quality of care to consider the quality of life, recognizing that this is unique for each individual. One key aspect is the importance of open conversations to determine what to expect from professional care. This aligns with the preferences expressed by older adults when discussing the quality of geriatric rehabilitation.

A strength of this study is that it was conducted with older adults currently undergoing rehabilitation, eliminating recall bias. Participants were allowed to generate quality ideas in multiple ways. First, they had the chance to formulate personal ideas in silence. These ideas were then shared within the group, supplemented with quality aspects derived from the literature^10^ and an interview^11^ study. By allowing participants first individually to reflect on the quality of rehabilitation, each participant had the opportunity to express their opinion before the discussion began. Additionally, the study was conducted across multiple rehabilitation centers with older adults with varying diagnoses to achieve results that are as generalizable as possible. Conversely, the study was conducted exclusively in rehabilitation institutions in the Netherlands. Countries with different rehabilitation care structures should critically assess the applicability of these quality ideas. However, these ideas are relevant to many healthcare institutions. Another limitation is that generalizability may be limited because we precluded participation of older adults with severe speech, language, or cognitive problems. However, we tried to include a diverse population regarding diagnoses, sex, and age. Lastly, a key strength of using this method is that the prioritization is supported by qualitative analysis of the discussions, providing a solid foundation for the results.

In conclusion, this nominal group technique yields high-quality topics prioritized by older adults in geriatric rehabilitation. This prioritization can help geriatric rehabilitation teams begin implementing quality improvement from the perspective of older adults. An individualized approach is essential in rehabilitation, as older adults have diverse preferences regarding responsibility and care, emphasizing the need for open communication and personalized support. Working with human beings involves listening to them. Future research is needed on how to implement these prioritized quality ideas in practice.

**Clinical messages**

- Actively involving older adults in identifying priorities for quality helps tailor geriatric rehabilitation to their needs and expectations.
- Clear communication, shared decision-making, and goal setting from the start of rehabilitation were prioritized to enhance older adults’ engagement and satisfaction.
- Organized, person-centred care—delivered by consistent and empathetic staff—contributes to trust, autonomy, and better rehabilitation experiences.
- Using prioritization methods such as the Nominal Group Technique can inform quality improvement strategies and implementation according to older adults’ perspectives.

## Supplemental Material

sj-xlsx-1-cre-10.1177_02692155251404003 - Supplemental material for Prioritizing quality of geriatric rehabilitation from the older adults’ perspective: A nominal group technique study Supplemental material, sj-xlsx-1-cre-10.1177_02692155251404003 for Prioritizing quality of geriatric rehabilitation from the older adults’ perspective: A nominal group technique study by Anne L Lubbe, Wim G Groen, Marjon van Rijn, Deborah C Mittelmeijer, Cees MPM Hertogh, Bianca M Buurman and Margriet C Pol in Clinical Rehabilitation
